# The Influence of Annealing on the Structural and Soft Magnetic Properties of (Fe_0.4_Co_0.6_)_79_Nb_3_B_18_ Nanocrystalline Alloys

**DOI:** 10.3390/ma11112171

**Published:** 2018-11-02

**Authors:** Man Zhu, Yang Fa, Lijuan Yao, Peng Tao, Zengyun Jian, Fang’e Chang

**Affiliations:** School of Materials Science and Chemical Engineering, Xi’an Technological University, Xi’an 710021, China; fayang2025@163.com (Y.F.); ylj8453@126.com (L.Y.); 18762794998@126.com (P.T.); jianzengyun@xatu.edu.cn (Z.J.); cfe.ch@163.com (F.C.)

**Keywords:** Fe-based nanocrystalline alloys, isothermal annealing, grain size, soft magnetic properties

## Abstract

The soft magnetic properties of Fe-based nanocrystalline alloys are determined by their grain size. In the present article, the (Fe_0.4_Co_0.6_)_79_Nb_3_B_18_ nanocrystalline alloys have been successfully prepared by isothermal annealing. The variation of soft magnetic properties as a function of annealing temperature and incubation time is investigated in detail. Two distinct crystallization behaviors were found for the (Fe_0.4_Co_0.6_)_79_Nb_3_B_18_ alloys. The initial nanocrystallization products comprise a mixture of *α*-Fe(Co), Fe_2_B, and Fe_23_B_6_-type crystalline metastable phases, and the final crystallization products are composed of *α*-Fe(Co), Fe_2_B, and Fe_3_B crystalline phases. The grain size decreases first and then increases with the increasing annealing temperature in the range of 764–1151 K, and a fine grain size with mean grain size of 12.7 nm can be achieved for alloys annealed at 880 K. As the annealing temperature increases from 764 K to 1151 K, the saturation magnetization increases first and then decreases without a significant increase of the coercivity. The alloys annealed at 880 K exhibit the optimized soft magnetic properties with high *M*_s_ of 145 emu g^−1^ and low *H*_c_ of 0.04 Oe.

## 1. Introduction

In 1988, Yoshizawa et al. [1] developed a new series of FeCuNbSiB nanocrystalline alloys with excellent magnetic properties. In 2013, Rizal et al. [2] artificially produced Fe-Co alloys with record high saturation magnetization (*M*_s_ > 240 emu/g). This group claimed that the increase of *M*_s_ is correlated with the increase of the lattice constant of the alloy. Thus, these Fe-based soft magnetic alloys have received great attention in the materials field [3,4,5]. The Fe-based nanocrystalline alloys with high performance have been served as the magnetic cores, sensors, and transformers in electrical industrial fields, and they are potential candidates to replace traditional soft magnetic alloys, such as silicon steel and ferrite. Generally speaking, metallic glass is in a thermodynamically metastable state. Upon heating, it would transform into a stable state by a crystallization process to form amorphous/nanocrystalline alloys. The soft magnetic properties in the Fe-based nanocrystalline alloys are determined by the type and grain size of the nano-scale precipitates. Nowadays, annealing crystallization is one of the key methods used to prepare Fe-based nanocrystalline alloys [6,7,8,9,10]. Therefore, it is of theoretical significance and engineering application value to explore the relationship between their magnetic properties and annealing process. Generally speaking, the grain size increases with increasing annealing temperature [11,12,13]. However, according to recent studies, as the annealing temperature increases, the grain size increases first, then decreases, and increases again in the Fe-based nanocrystalline alloys [14,15]. Xue et al. [14] pointed out that the grain size reaches the minimum value for the Fe_83_Nb_2_B_14_Cu_1_ alloys annealed at 813 K, while their saturation magnetization increases steadily from 1.4 T to 1.7 T with the increase of annealing temperature. Li et al. [15] pointed out that the Fe_73.37_Cu_0.92_Nb_2.9_Si_13.56_B_9.24_ nanocrystalline alloys with the finest grain size exhibited optimized magnetic properties. However, the experiments carried out by Jiang et al. [16] indicated that the grain size in the Fe_73.5_Cu_1_Nb_3_Si_13.5_B_9_ nanocrystalline alloys decreases slightly, and then increases with increasing annealing temperature, and the anomalous grain growth is ascribed to the different nucleation and volume diffusion rates at varied annealing temperatures. The variation of grain size as a function of annealing during the annealing process remains unclear, and it is quite difficult to determine the optimized annealing process for alloys with excellent soft magnetic properties. It is accepted that fine and well-distributed nanoscale precipitates within the amorphous matrix are of benefit to enhance the magnetic properties. Thus, it is of great importance to study the relationship between magnetic properties and the annealing process.

In this article, the aim is to explore the relationship between grain size and annealing temperature in the (Fe_0.4_Co_0.6_)_79_Nb_3_B_18_ alloys. In addition, the soft magnetic properties as a function of the kinds and size of the precipitates are also studied, which can provide theoretical guidance for the development of Fe-based nanocrystalline alloys with excellent soft magnetic properties.

## 2. Materials and Methods

Master alloy ingots with nominal composition of (Fe_0.4_Co_0.6_)_79_Nb_3_B_18_ were prepared by induction melting (SPG-40B high frequency induction heating equipment, Shenzhen, China) the mixture of pure Co (99.98 wt%), Fe (99.8 wt%), Fe-60 wt% Nb and Fe-17.5 wt% B master alloy. Then, the amorphous ribbons were prepared by melt-spinning method under high vacuum single-roller spinning equipment. During this process, the linear velocity and ejection pressure are 40 m/s and 20 kPa, respectively. The resulting ribbons have a thickness of 20–30 μm. Then, the isothermal annealing experiment was performed for the glassy ribbons sealed into quartz tubes under vacuum condition (4 × 10^−3^ Pa) in a resistance furnace. The annealing temperature (*T*_a_) was set as 764 K, 800 K, 880 K, 974 K, and 1151 K.

Phase constitution was identified by X-ray diffractometer (XRD; Bruker D8 advance, Bruker AXS GmbH, Karlsruhe, Germany) with Cu K*α* as a radiation (*λ* = 0.154056 nm) in steps of 0.02° in the 2*θ* = 30–120°. Thermal behaviors of the glassy ribbons were studied using differential scanning calorimetry (DSC; Mettler–Toledo TGA/DSC1, Mettler-Toledo International Inc., Zurich, Switzerland) at a continuous heating rate of 40 K min^−1^. Room-temperature magnetic hysteresis loops were measured using a vibrating sample magnetometer (VSM; Lake Shore 7410, Lake Shore Cryotronics, Columbus, OH, USA) under an applied magnetic field of 10,000 Oe.

## 3. Results and Discussion

### 3.1. Structure Identification and Thermal Properties in the As-Quenched (Fe_0.4_Co_0.6_)_79_Nb_3_B_18_ Alloys

**Figure 1:**
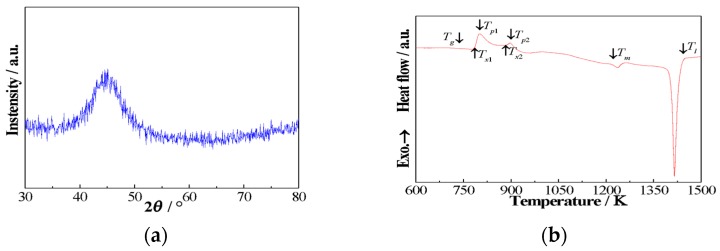
XRD pattern (**a**) and DSC heating curve (**b**) of the as-quenched (Fe_0.4_Co_0.6_)_79_Nb_3_B_18_ alloys.

Figure 1a shows the XRD pattern taken from the as-quenched (Fe_0.4_Co_0.6_)_79_Nb_3_B_18_ alloys. Only a wide diffraction peak in the 2*θ* region of 35–55° can be observed, and no sharp Bragg diffraction peaks corresponding to the crystalline phases are detected. This is the typical characteristic of amorphous alloys, indicating that a fully amorphous structure is obtained in the (Fe_0.4_Co_0.6_)_79_Nb_3_B_18_ alloys.

Figure 1b displays the DSC heating trace of the as-quenched (Fe_0.4_Co_0.6_)_79_Nb_3_B_18_ alloys with a constant heating rate of 40 K min^−1^. During the heating process, it undergoes a glass transition event followed by a supercooled liquid region, and then two-stage crystallization events are observed. The value of glass transition temperature (*T*_g_) is equal to 732 K, and the values of first crystallization onset temperature (*T*_x1_) and second crystallization onset temperature (*T*_x2_) are equal to 786 K and 887 K, respectively. The supercooled liquid region ∆*T*_x_ = *T*_x1_ − *T*_g_, is estimated to be 54 K. In the high-temperature region, there are two endothermic peaks. The values of solidus temperature (*T*_m_) and liquidus temperature (*T*_l_) are 1219 K and 1444 K, respectively. The parameter *T_rg_* (=*T*_g_/*T*_l_) is calculated to be 0.507. The results suggest that present (Fe_0.4_Co_0.6_)_79_Nb_3_B_18_ alloys possess high glass forming ability (GFA). The temperature intervals Δ*T*_N_ between two crystalline peaks, defined as *T*_x2_ − *T*_x1_, reach 101 K. It is reported that large Δ*T*_N_ is of benefit to the fabrication of amorphous/nanocrystalline alloys by precisely controlling the annealing process.

### 3.2. Structural Evolution in the Annealed (Fe_0.4_Co_0.6_)_79_Nb_3_B_18_ Alloys

**Figure 2:**
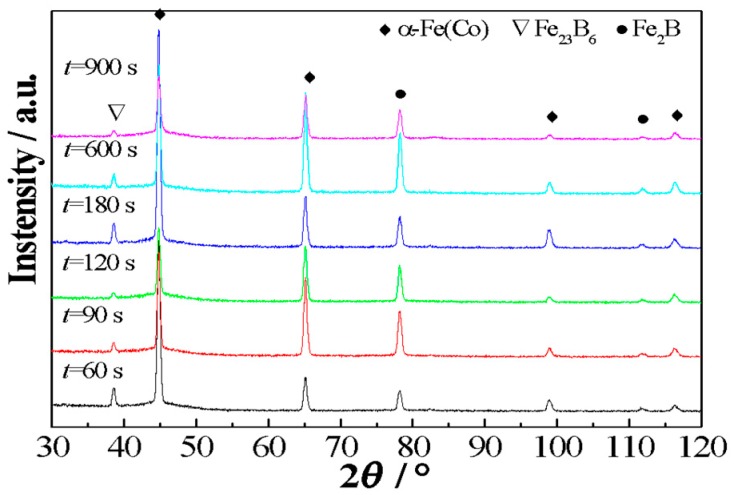
XRD patterns of the (Fe_0.4_Co_0.6_)_79_Nb_3_B_18_ alloys annealed at 764 K for varied times.

The (Fe_0.4_Co_0.6_)_79_Nb_3_B_18_ alloys were isothermally annealed at a temperature of *T*_a_ = 764 K, which is near *T*_x1_ between *T*_g_ and *T*_x1_. Figure 2 exhibits the XRD patterns of the (Fe_0.4_Co_0.6_)_79_Nb_3_B_18_ alloys annealed at 764 K for different incubation times. The crystallization products are composed of *α*-Fe(Co), Fe_2_B, and Fe_23_B_6_ phases for alloys annealed for 60 s. As the incubation time increases from 90 s to 900 s, the crystallization products still remain unchanged. The results indicate that increasing incubation time cannot change the crystallization process.

**Figure 3:**
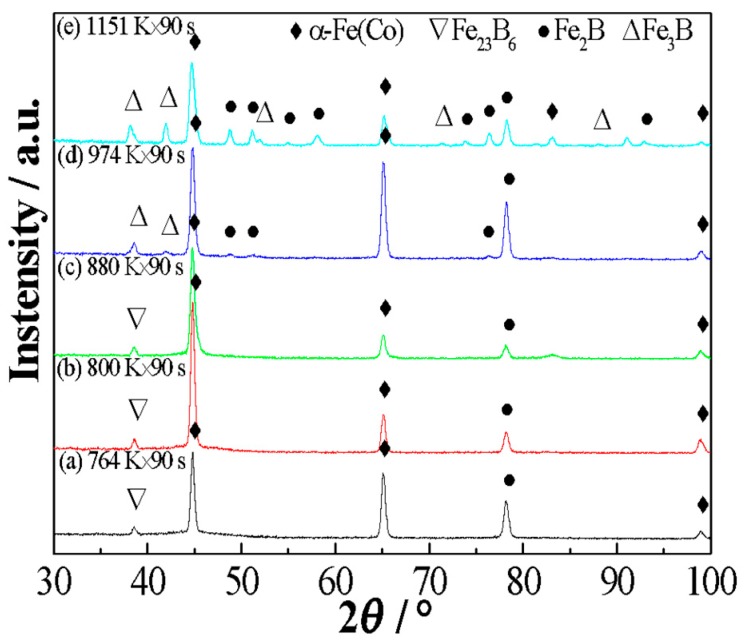
Structural evolution of the (Fe_0.4_Co_0.6_)_79_Nb_3_B_18_ alloys annealed at different temperatures for 90 s.

Figure 3 displays the XRD patterns of the (Fe_0.4_Co_0.6_)_79_Nb_3_B_18_ alloys annealed at 764 K, 800 K, 880 K, 974 K, and 1151 K for 90 s, respectively. For alloys annealed at 764 K, the crystallization products consist of a mixture of *α*-Fe(Co), Fe_23_B_6_, and Fe_2_B phases. The formation of a metastable Fe_23_B_6_ phase was previously reported in the FeNbB ternary alloys [3,17,18,19]. It has an *fcc* structure with a lattice constant of *a* = 1.076 nm. In this structure, the cubo-octahedra and the cubes formed by metal atoms are symmetrically connected with metalloid atoms. Thus, it would prevent long-range ordered diffusion during the solidification process, which favors the enhancement of GFA. For the alloys annealed at 800 K and 880 K, which range from *T*_x1_ to *T*_x2_, the crystallization products remain unchanged, and they are composed of *α*-Fe(Co), Fe_23_B_6_, and Fe_2_B phases. Because of the relatively short incubation time, the Fe_23_B_6_ phase is not completely decomposed. Thus, the existence of the metastable Fe_23_B_6_ phase still can be detected in the XRD patterns. Although the crystallization products remain unchanged, the corresponding width and intensity of the Bragg diffraction peaks change. This phenomenon suggests that the *α*-Fe(Co), Fe_23_B_6_, and Fe_2_B phases undergo grain growth processes, and their volume fraction increases. When the annealing temperature is larger than *T*_p2_, the crystallization products consist of *α*-Fe(Co), Fe_2_B, and Fe_3_B phases for alloys annealed at 974 K. The existence of Fe_2_B and Fe_3_B phases is attributed to the decomposition of the metastable Fe_23_B_6_ phase. As the annealing temperature is further increased to 1151 K, the crystallization products remain composed of *α*-Fe(Co), Fe_2_B, and Fe_3_B phases. The amount of relative diffraction peaks of Fe_2_B and Fe_3_B phases is increased, suggesting that the volume fractions of Fe_2_B and Fe_3_B phases are enhanced. Therefore, the crystallization processes in the (Fe_0.4_Co_0.6_)_79_Nb_3_B_18_ alloys can be expressed as:

Amorphous → Amorphous + *α*-Fe(Co) + Fe_23_B_6_ + Fe_2_B → *α*-Fe(Co) + Fe_2_B + Fe_3_B

The crystallinity refers to the degree of crystallization of amorphous alloys under different heat treatment processes. The crystallinity, *X*_c_, can be expressed as,

(1) $$X_{c}=\frac{W_{c}}{W_{c}+W_{A}},$$

where *W*_c_ and *W*_A_ represent the mass fraction of the crystalline phase and amorphous phase, respectively.

**Figure 4:**
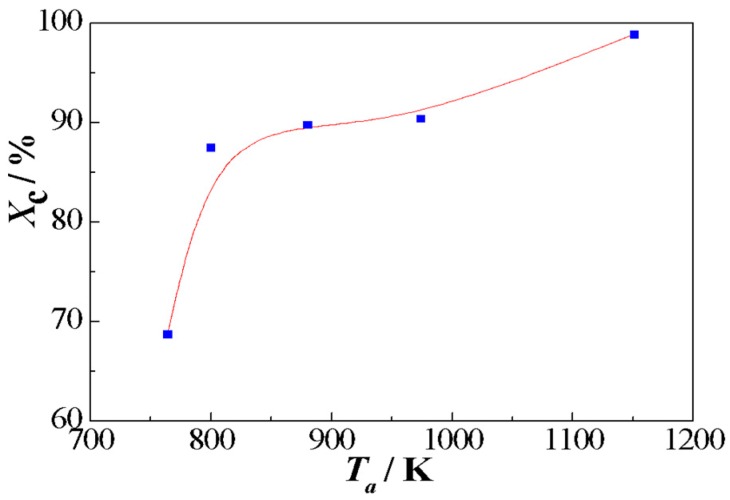
Variation of crystallinity as a function of annealing temperature for the (Fe_0.4_Co_0.6_)_79_Nb_3_B_18_ alloys.

Figure 4 shows the relationship between crystallinity *X*_c_ and annealing temperature *T*_a_. The value of *X*_c_ is equal to 68.69% for alloys annealed at 764 K. With the increase of the annealing temperature, the crystallinity increases steadily. As the annealing temperature is further increased to 1151 K, the value of *X*_c_ becomes 98.88%.

### 3.3. Variation of Grain Size and Annealing Temperature

According to the Scherrer Equation [6], the mean grain size (*D*) of the crystallite can be expressed as:

(2) $$\;D=\frac{k\lambda }{\beta \cos\theta }\;,\;$$

where *λ* is wavelength, *θ* is half the diffraction angle, *β* is full-width at half-maximum (FWHM) of the diffraction peak, and k is the constant (k = 0.89).

**Figure 5:**
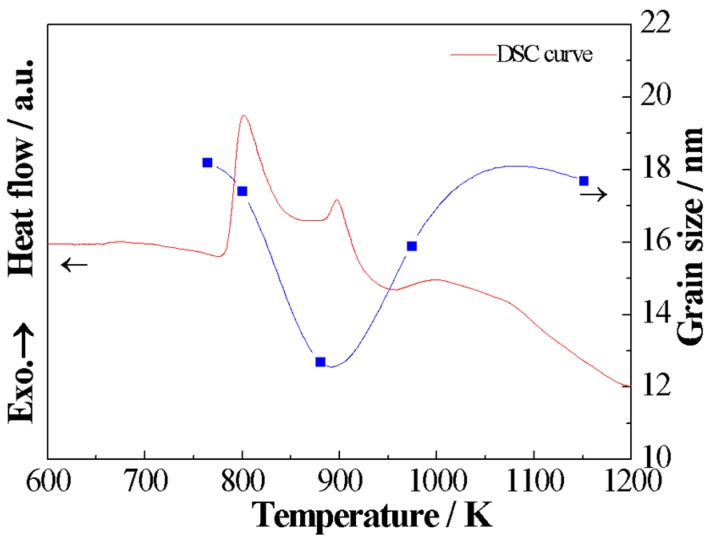
The relationship between mean grain size and annealing temperature for the (Fe_0.4_Co_0.6_)_79_Nb_3_B_18_ alloys.

The mean grain size (*D*) as a function of annealing temperature is plotted in Figure 5, and the corresponding DSC curve is also given. With the increase of the annealing temperature within the range 764–1151 K, the mean grain size decreases steadily and then increases. In other words, the grain size has obvious valley value with the change of the annealing temperature. The mean grain size (*D*) is equal to 18.2 nm and 17.7 nm for alloys annealed at 764 K and 1151 K, respectively. It reaches the minimum value of 12.7 nm for alloys annealed at 880 K.

The relationship between mean grain size and annealing temperature is in accordance with that predicted by theoretical analysis [20,21]. In other words, when the annealing temperature is equal to 0.6 *T*_m_, the mean grain size reaches the minimum value. It is directly related to the combined effect of a high nucleation rate and low grain growth rate. The crystallization process includes nucleation and grain growth processes. The nucleation rate *I*(*T_a_*) and grain growth rate *U*(*T_a_*) can be expressed as [20,22]:

(3) $$\;I\left(T_{a}\right)=I_{0}\exp\left(-\frac{\lambda ∆S_{m}^{f}}{R\tau^{2}{\left(1-\tau \right)}^{3}}\right)\exp\left(-\frac{∆E_{n}}{RT_{a}}\right),\;$$

(4) $$\;U\left(T_{a}\right)=U_{0}\left[1-\exp\left(-\frac{∆S_{m}^{f}\tau }{R\left(1-\tau \right)}\right)\right]\exp\left(-\frac{∆E_{g}}{RT_{a}}\right),\;$$

where $∆S_{m}^{f}$ is melting entropy, *τ* (=1 − *T_a_*/*T_m_*) the undercooling, *R* the gas constant, Δ*E_n_* the activation energy of nucleation, Δ*E_g_* the activation energy of diffusion, *T_a_* the annealing temperature, *T_m_* the melting temperature, and *I*_0_, *U*_0_, and *λ* are the constants.

**Figure 6:**
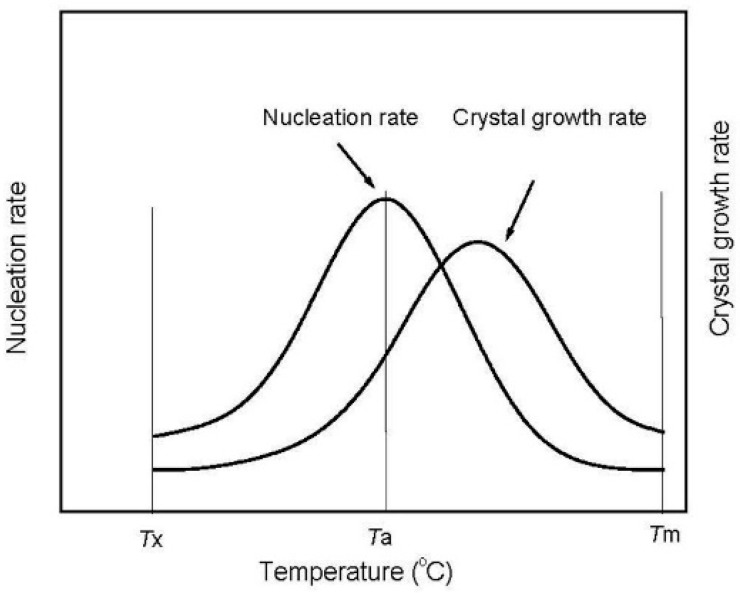
Schematic diagram showing the plots of nucleation rate and grain growth rate as a function of annealing temperature.

It can be found that the nucleation rate *I*(*T_a_*) is determined by *T_a_* and Δ*E_n_*, and grain growth rate *U*(*T_a_*) is associated with *T_a_* and Δ*E_g_*. The relationship between the nucleation rate and grain growth rate and annealing temperature is schematically plotted in Figure 6 [6], and *T_x_* and *T_m_* represent the first crystalline temperature and melting point, respectively. With the increase of *T_a_*, *I*(*T_a_*) and *U*(*T_a_*) increase first and then decrease. As the temperature increases from the first crystalline temperature to the temperature of the extreme value of the nucleation rate, the nucleation rate increases faster than the grain growth rate, and it reaches the extreme value quickly. Previous theoretical research confirmed that, when the annealing temperature is equal to approximately 0.6*T_m_*, a high nucleation rate and low grain growth rate can be achieved. Thus, it is reasonable to believe that the nanocrystalline alloys with fine grain sizes can be obtained for alloys annealed in the vicinity of 0.6*T_m_*.

### 3.4. Soft Magnetic Properties

**Figure 7:**
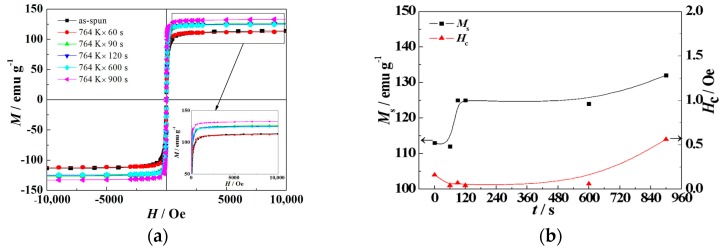
Magnetization hysteresis curves taken from the (Fe_0.4_Co_0.6_)_79_Nb_3_B_18_ alloys annealed at 764 K (**a**) and the variation of *M*_s_ and *H*_c_ as a function of incubation time *t* (**b**).

Figure 7a displays the room-temperature magnetization hysteresis curves (*M*–*H*) of the (Fe_0.4_Co_0.6_)_79_Nb_3_B_18_ alloys annealed at 764 K for 60–900 s, and the inset shows the enlarged image of the *M*–*H* curves. As can be seen from Figure 7a, the annealed alloys exhibit obvious soft magnetic properties without a significant increase of the coercivity. Figure 7b exhibits the relationship between saturation magnetization (*M*_s_) and coercivity (*H*_c_) and incubation time (*t*) in the (Fe_0.4_Co_0.6_)_79_Nb_3_B_18_ alloys. The *M*_s_ value is equal to 113 emu g^−1^ for the as-quenched (Fe_0.4_Co_0.6_)_79_Nb_3_B_18_ alloys. Annealing for 60 s does not result in a significant change in *M*_s_. As the incubation time further increases, the *M*_s_ value increases steadily. When the incubation time is equal to 900 s, the *M*_s_ value reaches the maximum value of 132 emu g^−1^. The increase of the *M*_s_ value is mainly determined by the grain size and volume fraction of the nano-scale precipitates. However, as the incubation time increases, the *H*_c_ value decreases slightly and then increases, and reaches 0.56 Oe for alloys annealed for 900 s. Short-time annealing benefits the reduction of internal stress, resulting in a slight decrease of *M*_s_. However, further annealing leads to an increase in grain size, resulting in high *M*_s_.

**Figure 8:**
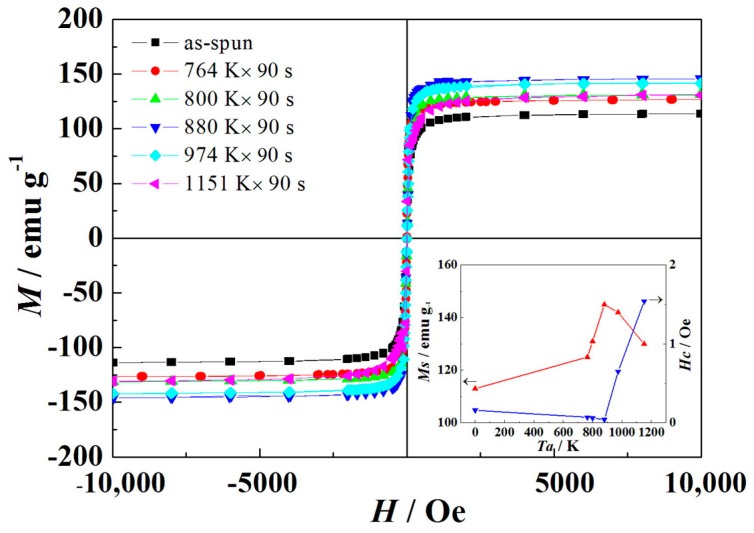
Room-temperature magnetization hysteresis curves for the (Fe_0.4_Co_0.6_)_79_Nb_3_B_18_ alloys annealed at different temperatures.

Figure 8 displays the *M*–*H* curves of the (Fe_0.4_Co_0.6_)_79_Nb_3_B_18_ alloys annealed at 764 K, 800 K, 880 K, 974 K, and 1151 K for 90 s, respectively. The insert clearly shows the relationship between *M*_s_ and *H*_c_ and annealing temperature. The VSM results indicate that the saturation magnetization (*M*_s_) increases first and then decreases with the increase of annealing temperature. The alloys annealed at 880 K exhibit the optimized soft magnetic properties with high *M*_s_ of 145 emu g^−1^ and low *H*_c_ of 0.04 Oe. The *α*-(Fe, Co) and Fe_23_B_6_ phases are ferromagnetic [17,23], while Fe_2_B or Fe_3_B phases belong to the hard magnetic phase [14]. During the annealing process, more and more short-range ordered clusters are formed within the amorphous matrix, and the coupling of these clusters leads to anisotropy. With the increase of annealing temperatures ranging from 764 K to 880 K, the crystallization products remain the same, while the mean grain size decreases. The ferromagnetic exchange between these *α*-Fe(Co) nanocrystals is enhanced, thus leading to an increase of *M*_s_. However, further increase of the annealing temperature results in coarse crystals with the precipitation of hard magnetic phases Fe_2_B or Fe_3_B [24,25]. As a result, the *M*_s_ value of the alloys is found to decrease.

According to the random anisotropy model (RAM) proposed by Hezer [26], the coercivity *H*_c_ is expressed as follows:

(5) $$\;H_{c}\approx p_{c}\frac{{K_{1}}^{4}D^{6}}{J_{s}A^{3}},\;$$

where *p*_c_ is the dimensionless pre-factor of the order of unity, *K*_1_ is the magneto-crystalline anisotropy, *A* is exchange stiffness, and *J*_s_ is saturation polarization.

The coercivity of the annealed (Fe_0.4_Co_0.6_)_79_Nb_3_B_18_ alloys is quite low. Similar results were also obtained in the Fe_74_Cu_0.8_Nb_2.7_Si_15.5_B_7_ alloys reported by Hoque et al. [27]. The coercivity of the annealed (Fe_0.4_Co_0.6_)_79_Nb_3_B_18_ alloys decreases first and then increases with the increase of annealing temperature. With the increase of annealing temperatures in the range of 764–880 K, the crystallization products remain the same, and mean grain size decreases, leading to a decrease of *H*_c_ value. However, a further increase of annealing temperature results in the formation of the Fe_2_B or Fe_3_B phases. It is reported that the *K*_1_ values for the Fe_2_B or Fe_3_B phases are equal to 430 kJ/m^3^ [24,27], which are larger than that of the *α*-Fe(Co) phase. Therefore, this increase would significantly increase the magneto-crystalline anisotropy, thus leading to an increase of *H*_c_ value.

## 4. Conclusions

(1) During the crystallization process in the (Fe_0.4_Co_0.6_)_79_Nb_3_B_18_ alloys, a mixture of *α*-Fe(Co), Fe_2_B, and Fe_23_B_6_ phases is first precipitated from the amorphous matrix, and then the formation of *α*-Fe(Co), Fe_2_B, and Fe_3_B phases occurs due to the decomposition of the metastable Fe_23_B_6_ phase.
(2) For alloys annealed at *T_g_*~*T_x_*_1_, as the incubation time increases, the saturation magnetization first remains unchanged, and then increases steadily, while the coercivity decreases first and then increases.
(3) The mean grain sizes of the annealed (Fe_0.4_Co_0.6_)_79_Nb_3_B_18_ alloys first decrease and then increase with the increase of annealing temperature. The mean grain size reaches the minimum value of 12.7 nm for alloys annealed at 880 K.
(4) The annealed (Fe_0.4_Co_0.6_)_79_Nb_3_B_18_ alloys exhibit excellent magnetic properties with high saturation magnetization and low coercivity. With the increase of annealing temperature, the saturation magnetization first increases and then decreases, while coercivity is not obviously enhanced. The alloys annealed at 880 K with the finest grain size exhibit the optimized soft magnetic properties.
